# Short- and Long-Term Effects of Ca(OH)_2_/ZnO Heteronanostructure on Photosystem II Function and ROS Generation in Tomato

**DOI:** 10.3390/ma18174078

**Published:** 2025-08-31

**Authors:** Panagiota Tryfon, Julietta Moustaka, Ilektra Sperdouli, Chrysanthi Papoulia, Eleni Pavlidou, George Vourlias, Ioannis-Dimosthenis S. Adamakis, Michael Moustakas, Catherine Dendrinou-Samara

**Affiliations:** 1 Laboratory of Inorganic Chemistry, Department of Chemistry, Aristotle University of Thessaloniki, 54124 Thessaloniki, Greece; tryfon.giota@gmail.com; 2 Department of Botany, School of Biology, Aristotle University of Thessaloniki, 54124 Thessaloniki, Greecemoustak@bio.auth.gr (M.M.); 3 Institute of Plant Breeding and Genetic Resources, Hellenic Agricultural Organization-Dimitra, 57001 Thessaloniki, Greece; ilektras@bio.auth.gr; 4 Department of Physics, Aristotle University of Thessaloniki, 54124 Thessaloniki, Greece; cpapoulia@physics.auth.gr (C.P.); elpavlid@auth.gr (E.P.); gvourlia@auth.gr (G.V.); 5 Section of Botany, Department of Biology, National and Kapodistrian University of Athens, 15784 Athens, Greece; iadamaki@biol.uoa.gr

**Keywords:** inorganic nanoparticles, post-synthesis, hetero-nanostructure, biostimulants, effective quantum yield of PSII, non-photochemical quenching, excess excitation energy, hydrogen peroxide, chlorophyll fluorescence imaging

## Abstract

Among different formations, inorganic/inorganic assemblies can be considered “two in one” systems offering collective and/or new physical-chemical properties and substantial activity. Herein, a post-synthetic approach involving the assembly through Van der Waals forces and/or hydrogen bonding of the preformed ZnO@OAm NPs and Ca(OH)_2_@OAm NPs of non-uniform sizes (9 nm and 27 nm, respectively), albeit coated with the same surfactant (oleylamine-OAm), is reported. The resulting semiconductor hetero-nanostructure (named CaZnO) has been physicochemically characterized. The X-ray diffraction (XRD) peaks correspond to both ZnO and Ca(OH)_2_, confirming the successful formation of a dual-phase system. Field emission scanning electron microscopy coupled with energy-dispersive spectroscopy (FESEM-EDS) of CaZnO indicated the formation of Ca(OH)_2_ NPs decorated with irregular-shaped ZnO NPs. The synthesized hetero-nanostructure was evaluated by assessing any negative effects on the photosynthetic function of tomato plants as well as for the generation of reactive oxygen species (ROS). The impact of the CaZnO hetero-nanostructure on photosystem II (PSII) photochemistry was evaluated under both the growth light intensity (GLI) and a high light intensity (HLI) at a short (90 min) and long (96 h) duration exposure. An enhancement of photosystem II (PSII) function of tomato plants by 15 mg L^−1^ CaZnO hetero-nanostructure right after 90 min was evidenced, indicating its potential to be used as a photosynthetic biostimulant, improving photosynthetic efficiency and crop yield, but pending further testing across various plant species and cultivation conditions.

## 1. Introduction

The challenge of feeding an estimated global population of 9.7 billion by 2050 presents significant pressure on current agricultural systems. To address this demand, it is crucial to develop and advance sustainable and eco-friendly agricultural technologies [1]. One promising approach is the application of nanomaterials, which has revolutionized agricultural practices by providing innovative solutions to enhance crop productivity and protection [2]. Inorganic nanoparticles (INPs) are particularly promising due to their unique physicochemical properties, since they improve plant health by controlling pathogens, increase nutrient availability, and enable controlled release of agrochemicals [3,4,5]. Metal-based INPs, which are often used as fertilizers and growth promoters, can enhance crop yields by 20% and reduce disease incidence by up to 50%. Furthermore, these INPs reduce nutrient leaching and enhance soil carbon sequestration, supporting sustainable agricultural practices. Their ability to boost plant defense mechanisms and nutrient uptake positions them as valuable tools in addressing critical agricultural challenges, with potential applications in nutrient management, disease control, and precision agriculture [6].

Among the various INPs, calcium hydroxide [Ca(OH)_2_ NPs] and zinc oxide (ZnO NPs) nanoparticles have gained significant attention fromus and others due to their versatility in applications such as dielectric materials, antimicrobial agents, cultural heritage preservation, and, notably, plant protection and growth enhancement. Their safety and stability further contribute to their widespread use [7,8,9,10].

Calcium is critical for plant health, playing an essential role in regulating photosynthesis [11,12]. It acts as a secondary messenger, enhancing stress tolerance and promoting growth and development [11,13,14]. In the oxygen-evolving complex (OEC) of photosystem II (PSII), calcium is a vital component of the Mn_4_CaO_5_ cluster, that catalyzes water oxidation [15,16]. Calcium ions are also crucial for photoprotection and repair of PSII under environmental stress [17]. Ca(OH)_2_ NPs exhibited strong antibacterial activity against both Gram-positive and Gram-negative bacteria [18,19], antifungal effects against *Botrytis cinerea* [20], and nematicidal properties against *Meloidogyne incognita* and *Meloidogyne javanica* [21]. Biocidal activities are primarily attributed to the generation of reactive oxygen species (ROS), inducing oxidative stress [22,23]. On the other hand, ZnO NPs play a crucial role in providing zinc (Zn), an essential micronutrient involved in key plant physiological processes, such as enzyme activation and photosynthesis [24]. ZnO NPs have been found to promote plant growth, enhance photosynthetic activity, and improve stress tolerance [25]. As a chemically and thermally stable semiconductor, ZnO can take various morphologies [26], and its antimicrobial activity has been evaluated across different microorganisms, showing size, shape, and structure-dependent efficacy [27]. Coated ZnO nanorods have been tested for antifungal activity against *Botrytis cinerea*, as well as pegylated ZnO nanoflowers as nematicides against *Meloidogyne javanica* [21,28]. Zinc oxide NPs improved drought stress resistance in *Arabidopsis thaliana* [29], while zinc, similarly to calcium, contributes to plant health by supporting chlorophyll synthesis and maintaining the proper functioning of the photosynthetic apparatus [30]. The effects of ZnO NPs have been concentration-dependent in species like *Arabidopsis thaliana* [31,32] and *Stevia rebaudiana* [33].

As a result of the above properties, the conjugation of ZnO NPs with Ca(OH)_2_ in different forms/structures represents a promising area of research due to their biocompatibility and combined antibacterial and structural properties. For instance, suspensions of Ca(OH)_2_-ZnO displayed good antifungal properties against *Penicillumoxalicum* and *Aspergillus niger* under simulated photoperiod conditions [34]. CaO-ZnO hierarchical heterostructures were found to have band gap restructuring due to n-n heterojunctions at high aspect ratios [35]. The chemical and crystallographic differences between the dissimilar materials give rise to the formation of structural and heterointerfacial complexities, that areof great significance from practical perspectives for any application [36]. There are also potential applications in agriculture with benefits in improving plant growth, enhancing nutrient availability, and mitigating stress conditions. In addition, studies on calcium-doped ZnO nanoparticles have shown significant improvements in seed germination, root and shoot growth, plant height, and leaf size in crops like maize (*Zea mays)* and wheat (*Triticum aestivum*) [37]. Nevertheless, the use of nanomaterials as nano-fertilizers to enhance crop productivity and sustainability has also raised concerns about their possible harmful impact on plants [38,39].

Foliar application of ZnO NPs enhanced tomato growth by increasing chlorophyll content and PSII activity [40]. Based on the concept of synergism and our recent promising results on oleylamine (OAm)-coated Ca(OH)_2_ and ZnO NPs in enhancing PSII function [40,41], further investigation of these NPs in a hetero-nanostructure formulation is assessed. In particular, primary ZnO@OAm NPs and Ca(OH)_2_@OAm NPsin a 1:1 ratio have been used for the formation of Ca(OH)_2_/ZnO hetero-nanostructures(named CaZnO) through a simple, mild, self-assembly approach based on the same coating and the different nanosizes of the initial NPs. The physicochemical characterization of the hetero-nanostructure was recorded via various techniques such as X-ray diffraction (XRD), Fourier transform infrared spectroscopy (FT-IR), UV–Visible spectroscopy (UV-Vis), thermogravimetric analysis (TGA), field emission scanning electron microscopy coupled with energy-dispersive spectroscopy (FESEM-EDS), dynamic light scattering (DLS), and ζ-potential measurements. Understanding the effect of CaZnO hetero-nanostructures on photosynthesis function is essential for evaluating their potential as biostimulants and their role in sustainable agricultural practices. Thus, in order to test the impact of the synthesized CaZnO hetero-nanostructure on plant growth, we evaluated the plant’s photosynthetic function for any cytotoxic effects as well as for the generation of reactive oxygen species (ROS), which can induce cell death, after short (90 min) and long-term (96 h) exposure. The impact of CaZnO hetero-nanostructure on PSII function in tomato (*Solanum lycopersicum* L.) plants was evaluated using chlorophyll fluorescence imaging analysis.

## 2. Materials and Methods

### 2.1. Materials

The reagents used for the synthesis of NPs and hetero-nanostructures were obtained and used without further purification. These included zinc (II) acetylacetonate hydrate [Sigma-Aldrich, St. Louis, MO, USA, *M* = 263.61 g mol^−1^, Zn(acac)_2_], calcium chloride (BDH Laboratory, Dubai, United Arab Emirates, *M* = 110.9 g mol^−1^, CaCl_2_), oleylamine (Merck, Darmstadt, Germany, *M* = 267.49 g mol^−1^, OAm), and ethyl alcohol (*M* = 46.07 g mol^−1^), dimethyl sulfoxide (DMSO), and chloroform (CHCl_3_).

### 2.2. Synthesis of Primary Nanoparticles

The primary coated metal-based INPs were conducted according to the following previously reported processes: ZnO@OAm NPs: Oleylamine-coated ZnO NPs with an irregular shape were synthesizedvia the solvothermal method [40]. Ca(OH)_2_@OAm NPs: Oleylamine-coated Ca(OH)_2_@OAm NPs with a hexagonal structure were synthesized based on a microwave-assisted process [41].

### 2.3. Synthesis of CaZnO Hetero-Nanostructure

Hetero-nanostructure was prepared utilizing the primary INPs described above. The as-prepared Ca(OH)_2_@OAm NPs (20 mg) were dissolved in DMSO (1 mL), and ZnO@OAm NPs (20 mg) were dissolved in chloroform (5 mL). Each mixture was sonicated separately for 20 min, then mixed and sonicated for 2 h at 25 °C in a packed tube. Following sonication, the solvents were allowed to evaporate gradually, resulting in the isolation of sample Ca(OH)_2_/ZnO NPs called CaZnO.

### 2.4. Physicochemical Characterization

The physicochemical properties of hetero-nanostructure CaZnO were characterized using various techniques. X-ray diffraction (XRD) analysis was conducted to ascertain the average crystalline size and structure, employing a Philips PW 1820 diffractometer across a 2θ range of 10 to 90°, utilizing monochromatized Cu Kα radiation (λ = 1.5406 Å). Thermal stability and organic content of the hetero-nanostructure were assessed by thermogravimetric analysis (TGA) using a SETA-RAM SetSys-1200, with a temperature increase from 30 °C to 800 °C at a rate of 10 °C per minute in a nitrogen environment. Microstructural analyses were conducted using a Field Emission Scanning Electron Microscope (FESEM), model JEOL JSM-7610F Plus, equipped with an integrated X-ray Energy Dispersive Spectrometer (EDS) and the AZTEC ENERGY advanced system from OXFORD for elemental analysis. Measurements were carried out using both secondary electron imaging (SEI) and backscattered electron composition imaging (COMPO) modes at an acceleration voltage of 15kV and a working distance of approximately 8 mm. Fourier-transform infrared spectroscopy (FT-IR) measurements were obtained on a Nicolet iS20 series spectrometer (Thermo Fisher Scientific, Waltham, MA, USA) equipped with a monolithic diamond ATR crystal (4000 − 450 cm^−1^). Optical characteristics were investigated in an ethanol-water solution, utilizing a Jasco V-750 UV-Vis spectrophotometer (Tokyo, Japan). Particle size distribution, polydispersity index (PDI), and surface charge (ζ potential) were determined through dynamic light scattering (DLS) analysis, conducted at 25 °C using a Malvern Zetasizer (Nano ZS apparatus and VASCO Flex™ Particle Size Analyzer NanoQ V2.5.4.0).

### 2.5. Plant Material and Growth Conditions

Tomato (*Solanum lycopersicum* L. cv Galli) plants were grown in a greenhouse with a day/night temperature of 24 ± 1/20 ± 1 °C, relative humidity of 60 ± 5/70 ± 5% day/night, and a14h photoperiod with photosynthetic photon flux density (PPFD) of 560 ± 20 μmol photons m^−2^ s^−1^.

### 2.6. Foliar Spraying of Hetero-Nanostructure on Tomato Plants

Tomato plants at leaf developmental stage 15, according to the BBCH numerical scale, were foliar sprayed each one with 15 mL of either 0 mg L^−1^ (control), 15 mg L^−1^, or 30 mg L^−1^ CaZnO hetero-nanostructure. Foliar application of NPs has proven advantages over other methods [40,41], while the choice of nanoparticle concentrations used was to match our previous studies [40,41] in order to enable direct comparisons. All treatments were performed with 3 plants and two independent biological replicates.

### 2.7. Chlorophyll Fluorescence Measurements

We used the method of chlorophyll fluorescence analysis, as described before [42], to evaluate the impact of the synthesized CaZnO hetero-nanostructure on tomato photosystem II (PSII). Measurements were conducted using the Imaging-PAM Fluorometer M-Series MINI-Version (Heinz Walz GmbH, Effeltrich, Germany). Tomato leaves were dark-adapted for 20 min before measuring the minimum (F*o*) and the maximum (F*m*) chlorophyll *a* fluorescence. The actinic light (AL) of 580 μmol photons m^−2^ s^−1^, representing the growth light intensity (GLI), and the high light intensity (HLI) of 1000 μmol photons m^−2^ s^−1^ were used to measure the steady-state photosynthesis (F*s*). Saturating pulses (SPs) every 20 s for 5 min, after application of theactinic light (AL), were used to obtain the maximum chlorophyll *a* fluorescence in the light (F*m*′), while the minimum chlorophyll *a* fluorescence in the light (F*o*′) was computed from the equation F*o*′ = F*o*/(F*v*/F*m* +F*o*/F*m*′) [43]. The Win V2.41a software (Heinz Walz GmbH, Effeltrich, Germany) was used to estimate the chlorophyll fluorescence parameters, which are described in detail in Table 1.

### 2.8. Imaging of Hydrogen Peroxide Generation

The generation of H_2_O_2_ in tomato leaflets was evaluated 90 min and 96 h after tomato plants were sprayed with 15 mg L^−1^, or 30 mg L^−1^ CaZnO hetero-nanostructure, as described previously [44]. Tomato leaves were incubated with 25 μM 2′, 7′-dichlorofluorescein diacetate (DCF-DA, Sigma Aldrich, Chemie GmbH, Schnelldorf, Germany) for 30 min in the dark and then observed with a Zeiss AxioImager Z2 epi-fluorescence microscope (Carl Zeiss MicroImaging GmbH, Göttingen, Germany) equipped with an AxioCam MRc5 digital camera [44].

### 2.9. Statistical Analysis

A two-way ANOVA analysis was conducted to evaluate statistically significant differences for every parameter with treatment (0 mg L^−1^ (control), 15 mg L^−1^, or 30 mg L^−1^ CaZnO hetero-nanostructure) and time (90 min or 96 h) as factors, followed by Tukey’s post hoc test. The data were checked for normality and homogeneity of variance with the Shapiro–Wilk test and Levene’s test, respectively. All statistical analyses were performed with R software (version 4.3.1, R Core Team, Vienna, Austria, 2023). Values were considered significantly different at *p* < 0.05. All results of the two-way ANOVAs are presented in Table S1.

## 3. Results

### 3.1. Physicochemical Characterization of the Hetero-Nanostructure

**Figure 1:**
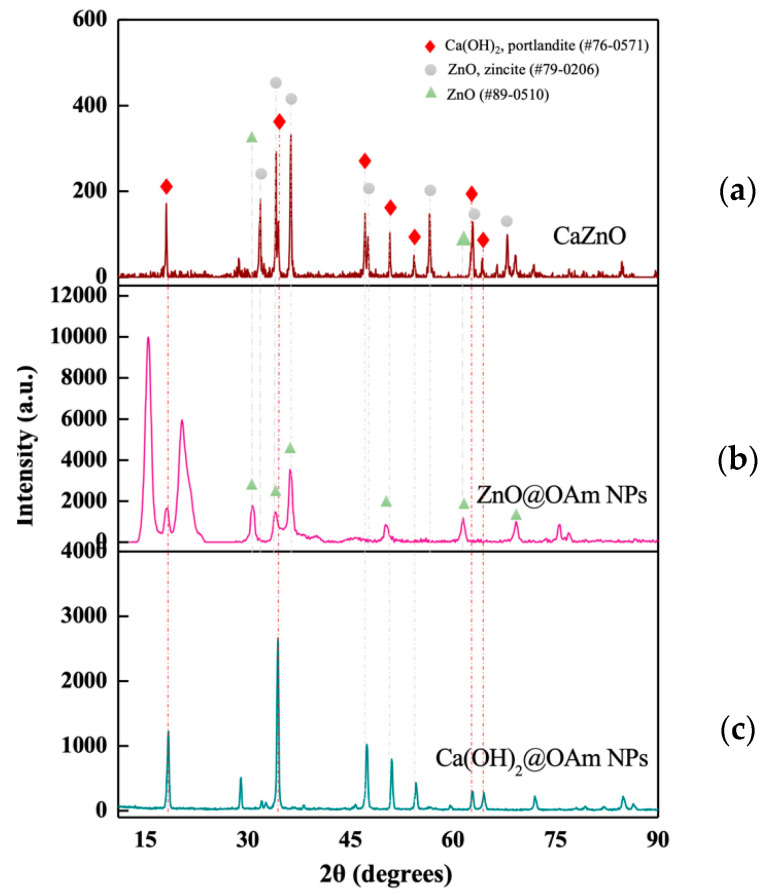
X-ray diffraction (XRD) pattern of the synthesized CaZnO hetero-nanostructure (**a**), compared with the XRD patterns of the primary nanoparticles ZnO@OAm NPs (**b**) and Ca(OH)_2_@OAm NPs (**c**).

The X-ray diffraction (XRD) for the CaZnO hetero-nanostructure (Figure 1a) reveals diffraction peaks corresponding to both zinc oxide (ZnO) and calcium hydroxide [Ca(OH)_2_], confirming the successful formation of a dual-phase system. The main peaks at 31.77° (100), 34.44° (002), and 36.4° (101) are characteristic of ZnO with zincite structure (pdf #79-0206), while additional peaks corresponding to Ca(OH)_2_ (pdf #76-0571) are also clearly visible, indicating the presence of the portlandite phase. However, unlike the initial ZnO@OAm NPs, whose XRD corresponds to a typical hexagonal wurtzite structure (space group P63mc (186), JCPDS card #89-0510) (Figure 1b), in the case of the hetero-nanostructure, an enhancement of the (002) peak relative intensity occurs, indicating a preferential growth orientation along the c-axis characteristic of the zincite structure. Additionally, the diffraction peaks of high intensities (at 2θ = 18–25) that are attributed to the well-crystallizedOAm on the surface of primary ZnO@OAm NPs are not detected in the hetero-nanostructure (Figure 1c).

**Figure 2:**
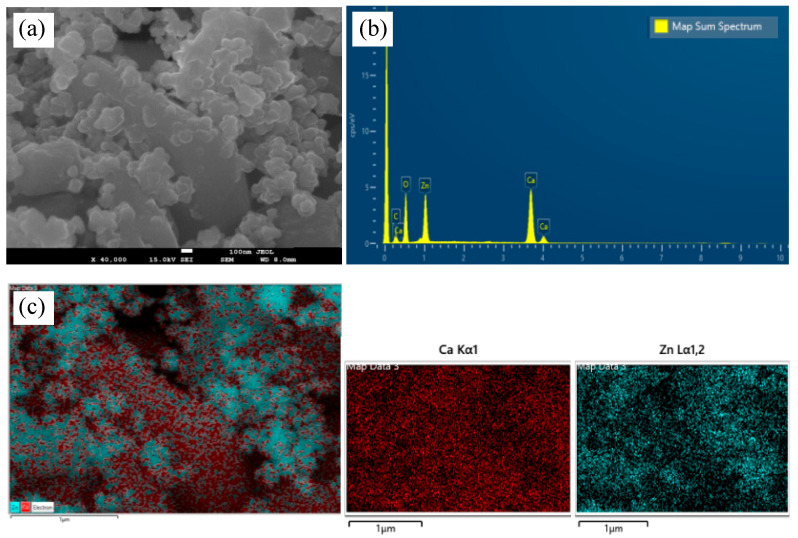
FESEM-EDS analysis of CaZnO hetero-nanostructure: (**a**) a SEM micrograph illustrating the microstructure, (**b**) EDS spectra identifying the elemental composition, and (**c**) SEM elemental mapping highlighting the spatial distribution of Ca and Zn elements. All micrographs are presented at a magnification scale of 1 µm.

The multimodal imaging and analysis of CaZnO hetero-nanostructure are presented in Figure 2, which demonstrates the material’s structure and elemental composition. Scanning electron microscope (SEM) images (Figure 2a) showed that irregular-shaped ZnO NPs were scattered across the surface of hexagonal Ca(OH)_2_ NPs. Figure 2b depicts the energy-dispersive X-ray spectroscopy (EDS) spectra, which confirms the presence of key constituent elements, with pronounced peaks for calcium (Ca) and zinc (Zn). Figure 2c illustrates SEM-based elemental mapping, providing a visual representation of the spatial distribution of Ca and Zn within the hetero-nanostructure. The elemental maps show distinct areas rich in each element, with red representing calcium and teal indicating Zn, thus verifying the heterogeneous composition of the CaZnO (Figure 2c). Materials were confirmed also through SEM image scanning in two different spectra (Figure S1).

The Fourier-transform infrared (FT-IR) spectroscopy profile for the CaZnO hetero-nanostructure, which will be referred to as CaZnO hereafter for simplicity, is presented in Figure S2. Analysis of the spectrum showed a broad peak at approximately 440 cm^−1^, which can be attributed to the Zn–O and Ca–O stretching vibrations. The strong peak at around 3640 cm^−1^ confirmed the presence of Ca(OH)_2_ due to its association with the –OH group. Moreover, peaks discerned at 2976 cm^−1^ and 2890 cm^−1^ corresponded to C–H stretching vibrations of OAm.

Thermogravimetric analysis (TGA) for the weight loss of organic coating (oleylamine, OAm) and thermal effect on CaZnO is presented in Figure S3. The TGA curve of CaZnO exhibits a distinct four-step decomposition profile. Initially, weight loss up to approximately 250 °C corresponds to the removal of physically adsorbed water. The second stage, from approximately 250 °C to 350 °C, involves desorption of surface hydroxyl groups and/or loosely bound organic molecules. The third and fourth stages, occurring between 350 °C and 650 °C, indicate progressive thermal degradation of the layered oleylamine coating. The total cumulative weight loss reached approximately11% *w*/*w* by 650 °C, confirming the presence and decomposition of the organic coating on CaZnO.

The UV-Vis absorption spectrum of CaZnO in aqueous solution (Figure S4) shows three distinct bands: two in the UV region, attributed to OAm (λ_max_ = 225 nm) and Ca(OH)_2_NPs (λ_max_ = 282 nm), and another in the visible region (λ_max_ = 377 nm), corresponding to ZnO NPs. Dynamic light scattering (DLS) analysis was conducted to determine the hydrodynamic size of the freshly prepared hetero-nanostructure (Figure S5a), revealing an average hydrodynamic diameter of 442 ± 2.3 nm with a polydispersity index (PDI) of 0.2. A relatively broad size distribution curve is evident in the graph. The ζ-potential was measured at-14.7 ± 1 mV (Figure S5b).

### 3.2. Impact of the Hetero-Nanostructureon the Allocation of the Absorbed Light Energy in Photosystem II

**Figure 3:**
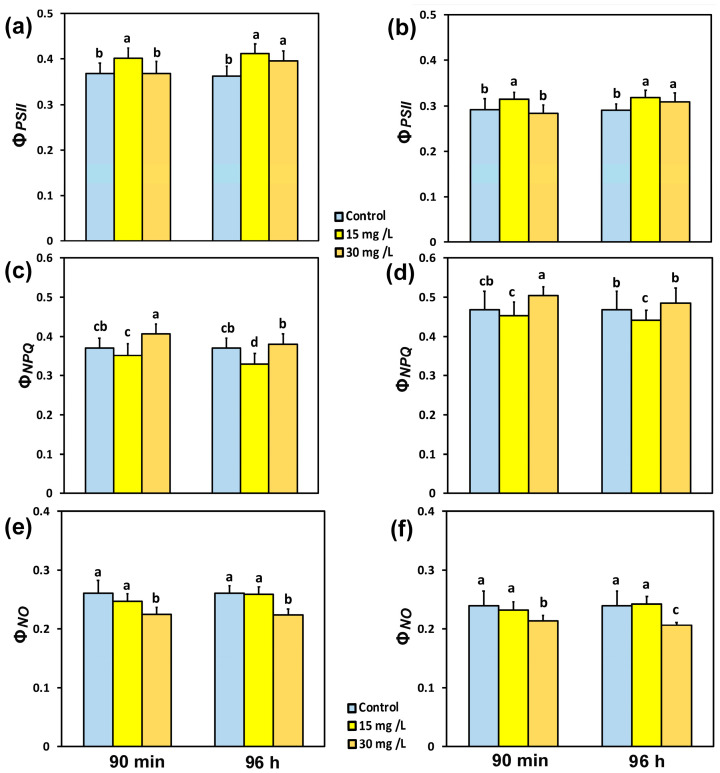
Light energy use efficiency in PSII. The effective quantum yield of PSII photochemistry (Φ_PSII_) (**a**,**b**), the quantum yield of regulated non-photochemical energy loss in PSII (Φ*_NPQ_*) (**c**,**d**), and the quantum yield of non-regulated energy loss in PSII (Φ*_NO_*) (**e**,**f**) measured at a growth light intensity of 580 μmol photons m^−2^ s^−1^ (**a**,**c**,**e**) and at the high light intensity of 1000 μmol photons m^−2^ s^−1^ (**b**,**d**,**f**) in tomato leaves 90 min and 96 h after exposure to 0 mg L^−1^ (control), 15 mg L^−1^ and 30 mg L^−1^ CaZnO. Standard deviations (SD) are shown by bars. Statistically significant differences (*p* < 0.05) are indicated by different lower-case letters (*n* = 6).

The absorbed light energy in PSII is portioned into photochemistry (Φ*_PSII_*), regulated loss as heat (Φ*_NPQ_*), and non-regulated loss (Φ*_NO_*). The yield of PSII photochemistry (Φ*_PSII_*), 90 min after exposure of tomato leaflets to 15 mg L^−1^ CaZnOincreased at both the GLI (9%) (Figure 3a) and the HLI (8%) (Figure 3b), while there was no change of Φ*_PSII_* with 30 mg L^−1^ CaZnO, compared to controls. Ninety-six hoursafter exposure of tomato leaflets to 15 mg L^−1^ CaZnO, Φ*_PSII_* increased by 12% at the GLI (Figure 3a) and by 9% at the HLI (Figure 3b), while with 30 mg L^−1^ CaZnO, it increased by 8% at the GLI (Figure 3a), and by 6% at the HLI (Figure 3b).

Ninety minutes after exposure of tomato leaflets to 30 mg L^−1^ CaZnO, the regulated non-photochemical energy loss in PSII (Φ*_NPQ_*) increased at the GLI by 9% (Figure 3c) and at the HLI by 7% (Figure 3d), while with 15 mg L^−1^ CaZnO, there were no significant changes in Φ*_NPQ_* at both light intensities. Ninety-six hours after exposure of tomato leaflets to 15 mg L^−1^ CaZnO, Φ*_NPQ_* decreased at the GLI by 11% (Figure 3c) and at the HLI by 6% (Figure 3d), while there were no significant changes in Φ*_NPQ_* at both light intensities with 30 mg L^−1^ CaZnO. The non-regulated loss (Φ*_NO_*), at both light intensities and time treatments, decreased by 30 mg L^−1^ CaZnO compared to the corresponding controls, while there were no changes in Φ*_NO_* with 15 mg L^−1^ CaZnO in comparison to controls (Figure 3e,f).

### 3.3. The Fraction of Open Photosystem II Reaction Centers and the Efficiency of PSII Reaction Centers Before and After Spraying with the Hetero-Nanostructure

**Figure 4:**
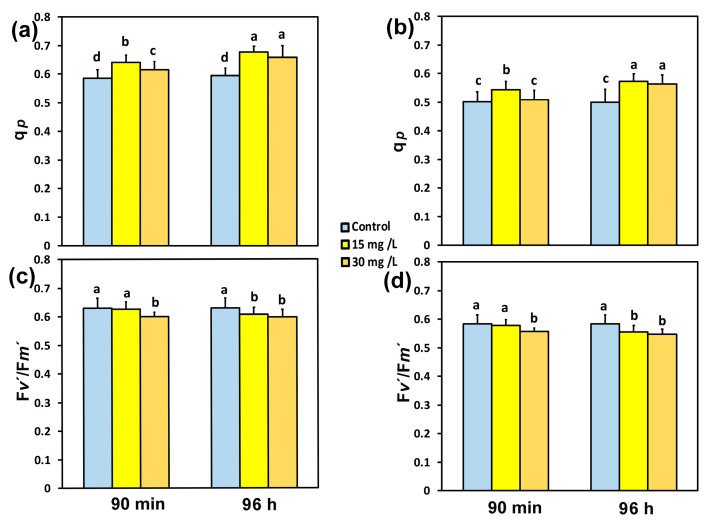
The fraction of open PSII reaction centers (RCs) (q*p*) (**a**,**b**) and theefficiency of the open PSII RCs (F*v*′/F*m*′) (**c**,**d**) measured at the growth light intensity of 580 μmol photons m^−2^ s^−1^ (**a**,**c**) and at the high light intensity of 1000 μmol photons m^−2^ s^−1^ (**b**,**d**) in tomato leaves 90 min and 96 h after exposure to 0 mg L^−1^ (control), 15 mg L^−1^, and 30 mg L^−1^ CaZnO. Standard deviations (SD) are shown by bars. Statistically significant differences (*p* < 0.05) are indicated by different lower-case letters (*n* = 6).

The fraction of reaction centers (RCs) that were open at PSII (q*p*) in tomato leaflets, 90 min after exposure to 15 mg L^−1^ CaZnO, increased by 9% at both the GLI (Figure 4a) and at the HLI (Figure 4b). At the same exposure time (90 min), the increase in q*p* in tomato leaflets by 30 mg L^−1^ CaZnO was 5% at the GLI (Figure 4a) and without any significant change at the HLI (Figure 4b). Ninety-six hours after exposure of tomato leaflets to 15 mg L^−1^ CaZnO, the fraction of open PSII reaction centers (q*p*) increased by 16% at the GLI (Figure 4a) and at the HLI by 14% (Figure 4b), while with 30 mg L^−1^ CaZnO, it increased by 13% at both the GLI (Figure 4a), and at the HLI (Figure 4b).

The efficiency of the open PSII RCs (F*v*′/F*m*′) did not change 90 min after exposure to 15 mg L^−1^ CaZnO at both the GLI (Figure 4c) and at the HLI (Figure 4d), while with 30 mg L^−1^ CaZnO, it decreased by 5% at both the GLI (Figure 4c) and at the HLI (Figure 4d). Ninety-six hours after exposure of tomato leaflets to 15 mg L^−1^ CaZnO, the efficiency of the open PSII RCs (F*v*′/F*m*′) decreased by 3% at the GLI (Figure 4c) and by 5% at the HLI (Figure 4d). At the same exposure time (96 h), F*v*′/F*m*′ decreased with 30 mg L^−1^ CaZnO by 5% at the GLI (Figure 4c) and by 6% at the HLI (Figure 4d).

### 3.4. The Photoprotective Heat Dissipation and the Electron Transport Rate in PSII Before and After Spraying with Hetero-Nanostructure

**Figure 5:**
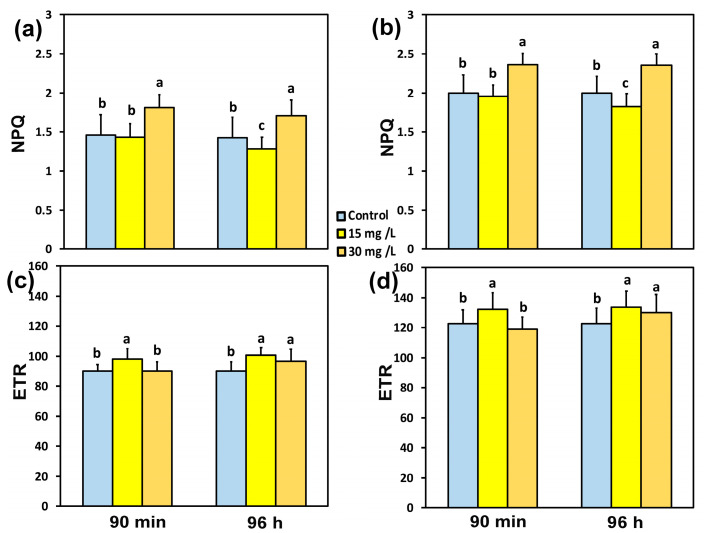
The non-photochemical quenching (NPQ) (**a**,**b**) and theelectron transport rate (ETR) (**c**,**d**) measured at the growth light intensity of 580 μmol photons m^−2^ s^−1^ (**a**,**c**) and at the high light intensity of 1000 μmol photons m^−2^ s^−1^ (**b**,**d**) in tomato leaves 90 min and 96 h after exposure to 0 mg L^−1^ (control), 15 mg L^−1^, and 30 mg L^−1^ CaZnO. Standard deviations (SD) are shown by bars. Statistically significant differences (*p* < 0.05) are indicated by different lower-case letters (*n* = 6).

The non-photochemical quenching (NPQ), which dissipates excess energy as heat, did not change 90 min after exposure to 15 mg L^−1^ CaZnO at both the GLI (Figure 5a) and at the HLI (Figure 5b), while with 30 mg L^−1^ CaZnO, it increased by 24% at the GLI (Figure 5a) and by 18% at the HLI (Figure 5b). After 96 h exposure to 15 mg L^−1^ CaZnO, NPQ decreased by 12% at the GLI (Figure 5a) and by 8% at the HLI (Figure 5b). At 96 h exposure to 30 mg L^−1^ CaZnO, NPQ increased by 17% at the GLI (Figure 5a) and by 18% at the HLI (Figure 5b).

The electron transport rate (ETR), 90 min after exposure of tomato leaflets to 15 mg L^−1^ CaZnO, increased at both the GLI (9%) (Figure 5c) and the HLI (8%) (Figure 5d), while there was no change in ETR at 30 mg L^−1^ CaZnO compared to controls. Ninety-six hours after exposure of tomato leaflets to 15 mg L^−1^ CaZnO, ETR increased by 12% at the GLI (Figure 5c) and at the HLI by 9% (Figure 5d), while with 30 mg L^−1^ CaZnO, it increased by 8% at the GLI (Figure 5c) and by 6% at the HLI (Figure 5d).

### 3.5. Impact of the Hetero-Nanostructure on the Excess Excitation Energy and the Excitation Pressure on PSII

**Figure 6:**
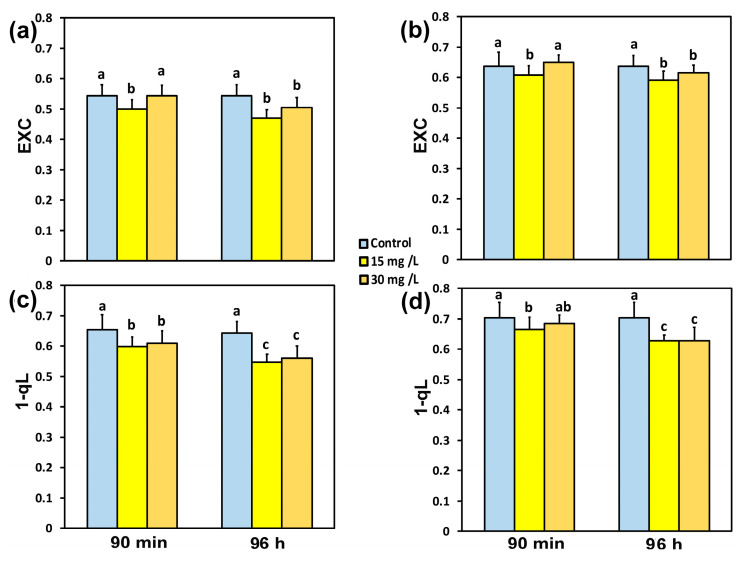
The excess excitation energy at PSII (EXC) (**a**,**b**) and the excitation pressure at PSII (1-qL) (**c**,**d**) measured at the growth light intensity of 580 μmol photons m^−2^ s^−1^ (**a**,**c**) and at the high light intensity of 1000 μmol photons m^−2^ s^−1^ (**b**,**d**) in tomato leaves 90 min and 96 h after exposure to 0 mg L^−1^ (control), 15 mg L^−1^, and 30 mg L^−1^ CaZnO. Standard deviations (SD) are shown by bars. Statistically significant differences (*p* < 0.05) are indicated by different lower-case letters (*n* = 6).

The excess excitation energy at PSII (EXC), 90 min after exposure of tomato leaflets to 15 mg L^−1^ CaZnO, decreased by 8% at the GLI (Figure 6a) and by 5% at the HLI (Figure 6b). Exposure of tomato leaflets to 30 mg L^−1^ CaZnO for 90 min did not have any influence on the EXC at both the GLI (Figure 6a) and the HLI (Figure 6b). However, exposure of tomato leaflets to 30 mg L^−1^ CaZnO for 96 h decreased the EXC by 7% at the GLI (Figure 6a) and by 4% at the HLI (Figure 6b), while 15 mg L^−1^ CaZnO decreased the EXC by 13% at the GLI (Figure 6a) and by 7% at the HLI (Figure 6b).

The excitation pressure at PSII (1-qL) 90 min after exposure of tomato leaflets to 15 mg L^−1^ CaZnO decreased by 9% at the GLI (Figure 6c) and by 6% at the HLI (Figure 6d). Exposure of tomato leaflets to 30 mg L^−1^ CaZnO for 90 min decreased the excitation pressure (1-qL) by 7% at the GLI (Figure 6c), but there was no significant difference at the HLI (Figure 6d). Ninety-six hours after exposure of tomato leaflets to 15 mg L^−1^ CaZnO, the excitation pressure (1-qL) decreased by 16% at the GLI (Figure 6c) and by 11% at the HLI (Figure 6d), while with 30 mg L^−1^ CaZnO, the excitation pressure (1-qL) decreased by 14% at the GLI (Figure 6c) and by 11% at the HLI (Figure 6d).

### 3.6. Impact of the Hetero-Nanostructure on Hydrogen Peroxide Production

**Figure 7:**
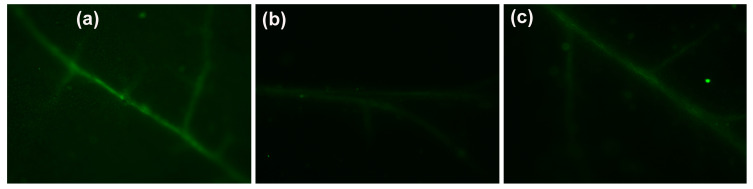
Imaging of H_2_O_2_ production in tomato leaves 90 min after their exposure to 0 mg L^−1^ (control) (**a**), 15 mg L^−1^ (**b**), and 30 mg L^−1^ (**c**); CaZnO. The light green color denotes H_2_O_2_ generation.

Hydrogen peroxide production in tomato leaves 90 min after exposure to 30 mg L^−1^ CaZnO (Figure 7c) decreased compared to control leaves (Figure 7a), while after 90 min exposure to 15 mg L^−1^ CaZnO, it was even less and hardly detectable (Figure 7b). H_2_O_2_ production was localized mainly in leaf veins, being visible as green fluorescence in tomato leaves.

### 3.7. Hormetic Responses of the Effective Quantum Yield of PSII Photochemistryto Hetero-Nanostructure

**Figure 8:**
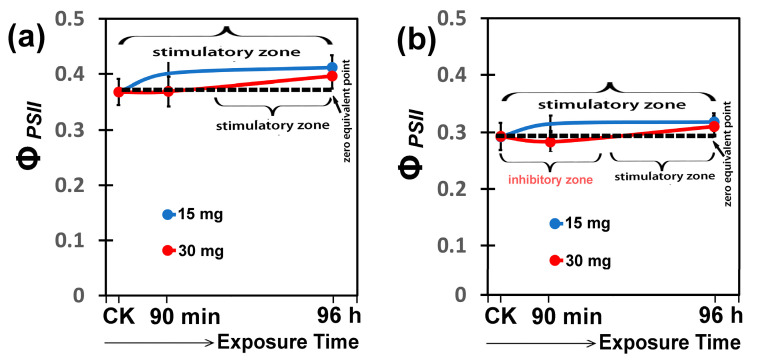
The J-shaped response curves of the quantum yield of PSII photochemistry (Φ*_PSII_*) to 15 mg L^−1^ or 30 mg L^−1^ CaZnO hetero-nanostructure, measured at the growth light intensity of 580 μmol photons m^−2^ s^−1^ (**a**) and at the high light intensity of 1000 μmol photons m^−2^ s^−1^ (**b**); of tomato leaves, 90 min and 96 h after exposure to 0 mg L^−1^ (CK, control), 15 mg L^−1^, or 30 mg L^−1^ CaZnO.

A stimulation of Φ*_PSII_* at both light intensities was observed with 15 mg L^−1^ CaZnO hetero-nanostructure immediately after the spray (Figure 8a,b), while the non-significant inhibitory effect of Φ*_PSII_* after short-duration exposure with 30 mg L^−1^ CaZnO hetero-nanostructure was restored at longer-duration exposure, reaching at 96 h exposure the stimulation of 15 mg L^−1^ CaZnO hetero-nanostructure (Figure 8a,b).

## 4. Discussion

Beyond the first generation of INPs, a second generation of more advanced nanoarchitectures is recently under investigation by us and others [45,46]. These hybrid engineered nanomaterials, such as bimetallic, hetero-nanostructures, inorganic/organic nanocapsules, etc., combine different functionalities, emerging as multimodal agents with combined and/or new artificial properties [2,47].

In the current study, a symbiotic hetero-nanostructure of Ca(OH)_2_/ZnO NPs was synthesized for the first time. A post-synthetic approach involving preformed ZnO@OAm and Ca(OH)_2_@OAm NPs of non-uniform crystallite sizes, 9 nm and 40 nm, respectively, has been used as building moieties for the preparation of CaZnO hetero-nanostructure. Sonication and solvents of different polarity were implemented during the synthetic procedure, resulting in the hetero-nanostructure consisting of ZnO NPs randomly decorated onto the bigger Ca(OH)_2_ NPs. The assembly process took place by attractive Van der Waals forces and/or hydrogen bonding through evaporation and when the reduction in the available volume occurred. The employed post-synthetic approach has also proven effective for the synthesis of other hetero-nanocomposites, copper(I) oxide with nickel ferrite NPs (Cu_2_O@NiFe_2_O_4_ NCs) [45].

The XRD pattern confirmed the presence of both Ca(OH)_2_NPs and ZnO NPs, with some modifications compared to the individual INP patterns (Figure 1b,c). The peaks were shifted and displayed lower intensity, indicating the successful formation of a condensed hetero-nanostructure. Reduction in the intensity of the main peaks corresponding to ZnO and Ca(OH)_2_, while the intensity at the crystallographic plane (011) remains at 100%. Notably, the ZnO structure was transformed during the synthetic process to the zincite phase, which is concomitant to the increment of the 002 plane (Figure 1a). Moreover, the structure of CaZnO was confirmed by the FT-IR spectrum with peaks attributed the bond stress vibrations due to the presence of amines, hydroxyls, and metal-oxide bonds [48,49]. Meanwhile, relative intensities at CaZnO are reduced and/or shifted due to the bigger size and less intake of OAm in comparison with the primary NPs (Figure S2). The presence of OAm was also certified using TGA. However, TGA of CaZnO when compared with the TGA curves of the primary nanoparticles (Figure S3) reveals differences in thermal decomposition behavior that are closely related to variations in OAm content, particle size, and surface chemistry. Smaller particles, such as ZnO@OAm (9 nm), possess higher surface-to-volume ratios, leading to greater surfactant adsorption and, consequently, higher organic content (30% *w*/*w*) and faster decomposition rates at relatively lower temperatures. Conversely, larger or structurally different nanoparticles, such as Ca(OH)_2_@OAm or the CaZnO hetero-nanostructure, exhibit stronger surfactant-particle interactions (e.g., Van der Waals forces), resulting in more thermally stable coatings and higher decomposition temperatures.

The clustered ZnO@OAm NPs around the free surface of Ca(OH)_2_@OAm NPs aresupported by SEM images and mapping of the elements (Ca, Zn). The DLS graph appeared in a particle size within a satellite distribution curve, corroboratingthe different sizes of the particles. The increment of the hydrodynamic size of CaZnO (442 nm) compared to that of its individual NP components, 73 nm and 145 nm for ZnO@OAm NPs and Ca(OH)_2_@OAm NPs, respectively, illustrates the altered morphology and distribution of the hetero-nanostructure.

Decoration of Ca(OH)_2_@OAm NPs with ZnO@OAm NPs led to changes in the size, shape, and distribution of the resulting CaZnO hetero-nanostructure in solution that can influence their optical properties. Specifically, the peak at 377 nm (band gap 3.29 eV) aligns closely with previously reported ZnO@OAm NPs (band gap 3.14 eV), confirming the integration of ZnO into the heterostructure. The peak at 282nm (band gap 4.40 eV) corresponds to Ca(OH)_2_, almost similar to previously reported monodispersed Ca(OH)_2_ at 4.35 eV and as expected for a wide band gap semiconductor [19]. The absorption peaks at 225 nm (band gap 5.51 eV) and 282 nm (band gap 4.40 eV) are consistent with electronic transitions involving ligand-to-metal charge transfer (LMCT) and interactions between NPs and the OAm coating. Typically, smaller NPs tend to primarily absorb light, whereas larger particles are prone to increased light scattering, causing the broadening of absorption peaks and a redshift towards longer wavelengths [50]. Metal-based mesoporous materials, due to their semiconductor properties, high surface area, and tunable pore sizes, are critical in photoelectrochemical cells for photosynthesis [51,52]. Both Ca(OH)_2_ and ZnO NPs, mainly in nanostructure form, possess direct band gaps that facilitate efficient charge transfer processes [48]. Ca(OH)_2_conduction band (4.4 eV) is higher than that ofZnO (3.29 eV), promoting electron migration from Ca(OH)_2_ to ZnO. This alignment prolongs charge carrier lifetimes, enabling sustained ROS generation (e.g., hydroxyl and superoxide radicals), as very recently proposed for ZnO-TiO_2_ nanocomposites that possess different band gaps [53]. Additionally, we assume that the negative ζ-potential, such as the -14.7 mV of CaZnO, isless likely to be trapped by cell membranes, enabling better translocation within plant tissues [54]. For instance, in wheat and tomato plants, cationic INPs tend to interact strongly with negatively charged plant cell membranes, leading to reduced mobility [55,56]. The absorption and transport mechanism of negatively/positively/neutral nanoparticles is still unclear, as it is a case-by-case issue [54]. Highly negatively charged particles are generally less likely to penetrate plant tissues than neutral or positively charged ones. However, if they are small or moderate, as in our case, and are stabilized with organic coatings, they may move more slowly or become adsorbed to tissue surfaces, and they may still travel through apoplastic pathways. Also, repulsion prevents aggregation and impacts longer shelf life and better dispersion.

Chlorophyll *a* fluorescence results from absorbed light energy; although it is only 0.6–5% of the absorbed energy, it can be decoded in terms of photosynthetic function to acquire information about the partitioning of the absorbed light energy at PSII [57,58,59]. The absorbed light energy is distributed to photochemistry (Φ*_PSII_*), or to heat loss, termed regulated non-photochemical energy loss (Φ*_NPQ_*), and to nonregulated energy loss (Φ*_NO_*), which are, in total, equal to 1 [60,61].

The lower photochemical efficiency (Φ_PSII_) induced by 30 mg L^−1^ CaZnO, after 90 min exposure, compared to that by 15 mg L^−1^, at both light intensities (Figure 3a,b), was overcompensated by the increased regulated non-photochemical energy loss in PSII (Φ*_NPQ_*) (Figure 3c,d) that resulted in lower non-regulated loss (Φ*_NO_*) (Figure 3e,f). A decreased Φ*_NO_* is regarded to be related to a decreased amount of singlet excited state of oxygen (^1^O_2_) generation [62,63,64,65]. ^1^O_2_ is highly reactive and damaging, formed through the interaction of molecular O_2_ with the excited triplet state of chlorophyll (^3^Chl*) [66,67,68,69,70].

The absorption of excess light that cannot be utilized for photochemistry must be dissipated harmlessly by the mechanism of non-photochemical quenching (NPQ) [66,71]. The mechanism of NPQ by dissipating the excess light energy as heat protects PSII from the formation of reactive oxygen species (ROS) that are harmful to plant cells [66,71,72,73]. Exposure of tomato leaves for 90 min and 96 h to 30 mg L^−1^ CaZnO stimulated NPQ at both the GLI and the HLI (Figure 5a,b), which resulted in decreased ^1^O_2_ as judged from Φ*_NO_* (Figure 3e,f). ^1^O_2_ produced by ^3^Chl* can further produce the other ROS, e.g., superoxide anion radical (O_2_^•^−) and H_2_O_2_ [74,75]. However, electron leakage to O_2_ which results in O_2_^•^−, which is being converted to H_2_O_2_, is the main pathway of H_2_O_2_ generation [76].

The induction of NPQ after 90 min exposure to 30 mg L^−1^ CaZnO, at both the GLI and the HLI, did not result in the down-regulation of PSII (Φ*_PSII_*), compared to controls, as observed under mild heat stress [72]. In contrast, the induction of NPQ 96 h after exposure of tomato leaves to 30 mg L^−1^ CaZnO resulted in an increased Φ*_PSII_* compared to controls (Figure 3a,b). The lower Φ*_PSII_* developed after 90 min exposure to 30 mg L^−1^ CaZnO, compared to that by 15 mg L^−1^, (Figure 3a,b), also resulted in a lower ETR (Figure 5c,d). This lower Φ*_PSII_* was due to the decreased efficiency of the open PSII reaction centers (RCs) (F*v*′/F*m*′) (Figure 4c,d), and not to the decreased fraction of the open PSII RCs (q*p*) (Figure 4a,b). Ninety-six hours after exposure of tomato leaflets to both CaZnO concentrations and light intensities, the quantum yield of PSII photochemistry (Φ_PSII_) increased (Figure 3a,b), despite the lower efficiency of the open PSII RCs (Figure 4c,d). This increased Φ*_PSII_* was due to the increased fraction of open PSII RCs (Figure 4a,b).

Exposure of tomato leaves for 90 min to 30 mg L^−1^ CaZnO resulted in decreased ^1^O_2_ generation, as estimated from Φ*_NO_*, (Figure 3e,f), but at the same time H_2_O_2_ production increased (Figure 6c), compared to 15 mg L^−1^ CaZnO (Figure 6b). Since ^1^O_2_ is formed by energy transfer, while H_2_O_2_ by electron transport, it seems probable that their signaling action sometimes can antagonize each other [77,78,79].

It can be concluded that exposure of tomato leaflets to 15 mg L^−1^ CaZnO seems to be more advantageous on PSII function compared to 30 mg L^−1^, by exerting earlier (90 min) its positive effect on PSII. However, PSII function was stimulated by both concentrations after 96 h exposure. The earlier beneficial effect on PSII function by 15 mg L^−1^ CaZnO was marked also by the lower excess excitation energy at PSII (EXC) measured at both light intensities after 90 min exposure to CaZnO (Figure 6a,b). The decreased excess excitation energy at PSII (EXC) after 90 min exposure to 15 mg L^−1^ CaZnO was correlated to the increased PSII quantum efficiency. This could probably be triggered by the modification of H_2_O_2_ homeostasis observed at the same period (Figure 7b). Hydrogen peroxide is the most stable ROS that can act as a long-distance signaling molecule and mediate plant responses to any changes in homeostasis [77,80,81,82,83]. Foliar-sprayed CaZnO developed a more oxidized redox state of the plastoquinol pool (q*p*) (Figure 4a,b), enhancing PSII quantum efficiency (Figure 3a,b) and modulating hydrogen peroxide generation (Figure 7). Redox regulation plays a crucial role in orchestrating signaling networks, incorporating those containing H_2_O_2_ [84]. Regulation of redox homeostasis enhances stress tolerance responses [85].

The response of Φ*_PSII_* to 15 mg L^−1^ and 30 mg L^−1^ CaZnO hetero-nanostructure looks like a hormetic response. Hormesis is termed the beneficial effect to an organism after exposure to a smalldose of an external factor that is followed by a negative effect at a larger dose of the same factor [86,87,88]. Zn-based NPs are used as nano-fertilizers to improve crop productivity by increasing photosynthetic function through improving light energy use efficiency, thus enhancing electron transport and elevating biomass crop production [89,90]. Photosystem II quantum efficiency of tomato plants increased significantly after 30 min exposure to 15 mg L^−1^ ZnO@OAm NPs [40] and after 90 min exposure to 15 mg L^−1^ CaZnO hetero-nanostructure (reported here), but not after 72 h exposure to 15 mg L^−1^ Ca(OH)_2_@OAm NPs [41]. Nanoparticle formulation, shape, size, and concentration play a critical role in the mechanism of their action and their impact on plant health status and ROS production [91,92,93,94]. It can be concluded from our results that chlorophyll *a* fluorescence imaging analysis and also multispectral polarimetric imaging can serve as non-invasive tools to monitor nanoparticle-induced stress in crops, reflecting changes in plant health status and ROS production mechanisms [79,95].

## 5. Conclusions

Inorganic nano-assemblies constitute a challenging feature for addressing modern agricultural aspects by improving crop productivity and sustainability. Ideally, materials with multifaceted properties that benefit plants can reduce environmental impacts. That basis was the motivation for the present study, and a novel “two-in-one” Ca(OH)_2_/ZnO hetero-nanostructure was successfully synthesized using a simple post-synthetic approach.

The CaZnO hetero-nanostructure consisted of hexagonal Ca(OH)_2_ NPs decorated with irregularly shaped ZnO NPs with a mean hydrodynamic size of 442 nm and a negative ζ-potential beneficial for translocation within plant tissues. Current properties provide them with potential in applications that require efficient electron movement, such as photoelectrochemical cells used in photosynthesis enhancement. The enhancement of PSII function by the CaZnO hetero-nanostructure, right after 90 min, indicates their potential to be used as photosynthetic bio-stimulants to enhance crop yields, pending further testing on other plant species. Furthermore, the natural abundance and non-toxicity of Ca(OH)_2_ and ZnO provide the bioavailability of nutrients, leading to better overall plant health.

## Figures and Tables

**Table 1:** Definitions of the chlorophyll fluorescence parameters used in the experiments.

Parameter	Definition	Calculation
*Fo*	Minimum chlorophyll *a* fluorescence in the dark-adapted leaf (PSII centers open)	Obtained by applying measuring photon irradiance of 1.2 μmol photons m^−2^ s^−1^
*Fm*	Maximum chlorophyll *a* fluorescence in the dark-adapted leaf (PSII centers closed)	Obtained with a saturating pulse (SP) of 6000 μmol photons m^−2^ s^−1^
*Fo*′	Minimum chlorophyll *a* fluorescence in the light-adapted leaf	It was computed by the Imaging Win software V2.41a (Heinz Walz GmbH, Effeltrich, Germany) as F*o*′ = F*o*/(F*v*/F*m* + F*o*/F*m*′)
*Fm*′	Maximum chlorophyll *a* fluorescence in the light-adapted leaf	Measured with saturating pulses (SPs) every 20 s for 5 min after application of the actinic light (AL) of 580 μmol photons m^−2^ s^−1^ or 1000 μmol photons m^−2^ s^−1^
*Fs*	Steady-state photosynthesis	Measured after 5 min illumination time before switching off the actinic light (AL) of 580 μmol photons m^−2^ s^−1^ or 1000 μmol photons m^−2^ s^−1^
Φ*_PSII_*	Effective quantum yield of PSII photochemistry	(F*m*′ − F*s*)/F*m*′
Φ*_NPQ_*	Quantum yield of regulated non-photochemical energy loss in PSII	F*s*/F*m*′ − F*s*/F*m*
Φ*_NO_*	Quantum yield of non-regulated energy loss in PSII	F*s*/F*m*
F*v*′/F*m*′	Efficiency of the open PSII reaction centers	(F*m*′ − F*o*′)/F*m*′
ETR	Electron transport rate	Φ*_PSII_* × PAR × c × abs, where PAR is the photosynthetically active radiation, c is 0.5, and abs is the total light absorption of the leaf taken as 0.84
q*p*	Photochemical quenching, representing the redox state of quinone A (Q_A_), or in other words the fraction of open PSII reaction centers based on the “puddle” model for the photosynthetic unit	(F*m*′ − F*s*)/(F*m*′ − F*o*′)
NPQ	Non-photochemical quenching reflecting the dissipation of excitation energy as heat	(F*m −* F*m*′)/F*m*′
EXC	Excess excitation energy	(1 − q*p*) × F*v*′/F*m*′
1-qL	The fraction of closed PSII reaction centers based on the “lake” model for the photosynthetic unit	1 − (q*p* × F*o*′/F*s*)

## Data Availability

The original contributions presented in this study are included in the article/Supplementary Materials. Further inquiries can be directed to the corresponding author.

## Supplementary Materials

The following supporting information can be downloaded at https://www.mdpi.com/article/10.3390/ma18174078/s1: Table S1: Results of two-way ANOVAs; Figure S1: Scanning electron microscopy (SEM) image of CaZnOhetero-nanostructure; Figure S2:Fourier-transform infrared (FT-IR) spectrum; Figure S3:Thermogravimetric analysis (TGA) curves; Figure S4: UV-Vis absorbance spectra of CaZnOhetero-nanostructure; Figure S5: Dynamic light scattering (DLS) analyses of CaZnOhetero-nanostructure.

## References

[1] Shang Y., Hasan K., Jalal A.G., Li M., Yin H., Zhou J. (2019). Applications of nanotechnology in plant growth and crop protection: A review. Molecules.

[2] Mohammadi S., Jabbari F., Cidonio G., Babaeipour V. (2024). Revolutionizing agriculture: Harnessing nano-innovations for sustainable farming and environmental preservation. Pestic. Biochem. Physiol..

[3] Yusuf A., Almotairy A.R.Z., Henidi H., Alshehri O.Y., Aldughaim M.S. (2023). Nanoparticles as drug delivery systems: A review of the implication of nanoparticles’ physicochemical properties on responses in biological systems. Polymers.

[4] Kanakari E., Dendrinou-Samara C. (2023). Fighting phytopathogens with engineered inorganic-based nanoparticles. Materials.

[5] Carrillo-Lopez L.M., Villanueva-Verduzco C., Villanueva-Sánchez E., Fajardo-Franco M.L., Aguilar-Tlatelpa M., Ventura-Aguilar R.I., Soto-Hernández R.M. (2024). Nanomaterials for plant disease diagnosis and treatment: A review. Plants.

[6] Francis D.V., Abdalla A.K., Mahakham W., Sarmah A.K., Ahmed Z.F.R. (2024). Interaction of plants and metal nanoparticles: Exploring its molecular mechanisms for sustainable agriculture and crop improvement. Environ. Int..

[7] European Food Safety Authority (EFSA) (2020). Outcome of the Consultation with Member States and EFSA on the Basic Substance Application for Approval of Calcium Hydroxide for the Extension of Use in Plant Protection As a Fungicide in Grapevine and Peach, and As Insecticide in Grapevine, Plum, Peach, Apricot, Apple, Pear, Almond and Strawberry.

[8] U.S. Food and Drug Administration (FDA) GRAS Notice. https://www.fda.gov/food/generally-recognized-safe-gras/gras-notice-inventory.

[9] Zhu J., Zhang P., Ding J., Dong Y., Cao Y., Dong W., Zhao X., Li X., Camaiti M. (2021). Nano Ca(OH)_2_: A review on synthesis, properties, and applications. J. Cult. Herit..

[10] Hamada A.M., Radi A.A., Al-Kahtany F.A., Farghaly F.A. (2024). A review: Zinc oxide nanoparticles: Advantages and disadvantages. J. Plant Nutr..

[11] Hepler P.K. (2005). Calcium: A central regulator of plant growth and development. Plant Cell.

[12] Verret F., Wheeler G., Taylor A.R., Farnham G., Brownlee C. (2010). Calcium channels in photosynthetic eukaryotes: Implications for evolution of calcium-based signalling. New Phytol..

[13] Ghosh S., Bheri M., Bisht D., Pandey G.K. (2022). Calcium signaling and transport machinery: Potential for development of stress tolerance in plants. Curr. Plant Biol..

[14] Gupta S., Kaur N., Kant K., Jindal P., Ali A., Naeem M. (2023). Calcium: A master regulator of stress tolerance in plants. S. Afr. J. Bot..

[15] Yocum C.F. (2008). The calcium and chloride requirements of the O_2_ evolving complex. Coord. Chem. Rev..

[16] Haddy A., Beravolu S., Johnston J., Kern H., McDaniel M., Ore B., Reed R., Tai H. (2024). Exploring the interdependence of calcium and chloride activation of O_2_ evolution in photosystem II. Photosynth. Res..

[17] Yang S., Wang F., Guo F., Meng J.J., Li X.G., Wan S.B. (2015). Calcium contributes to photoprotection and repair of photosystem II in peanut leaves during heat and high irradiance. J. Integr. Plant Biol..

[18] Karthik K., Dhanuskodi S., Gobinath C., Prabukumar S., Sivaramakrishnan S. (2017). Dielectric and antibacterial studies of microwave-assisted calcium hydroxide nanoparticles. J. Mater. Sci. Mater. Electron..

[19] Harish, Kumari S., Parihar J., Akash, Kumari J., Kumar L., Debnath M., Kumar V., Mishra R.K., Gwag J.S. (2022). Synthesis, characterization, and antibacterial activity of calcium hydroxide nanoparticles against gram-positive and gram-negative bacteria. ChemistrySelect.

[20] Tryfon P., Kamou N.N., Mourdikoudis S., Vourlias G., Menkissoglu-Spiroudi U., Dendrinou-Samara C. (2023). Microwave-mediated synthesis and characterization of Ca(OH)_2_ nanoparticles destined for geraniol encapsulation. Inorganics.

[21] Tryfon P., Antonoglou O., Vourlias G., Mourdikoudis S., Menkissoglu-Spiroudi U., Dendrinou-Samara C. (2019). Tailoring Ca-based nanoparticles by polyol process for use as nematicidals and pH adjusters in agriculture. ACS Appl. Nano Mater..

[22] Sanvicens N., Marco M.P. (2008). Multifunctional nanoparticles--properties and prospects for their use in human medicine. Trends Biotechnol..

[23] Samanta A., Podder S., Ghosh C.K., Bhattacharya M., Ghosh J., Mallik A.K., Dey A., Mukhopadhyay A.K. (2017). ROS mediated high anti-bacterial efficacy of strain tolerant layered phase pure nano-calcium hydroxide. J. Mech. Behav. Biomed. Mater..

[24] Faizan M., Faraz A., Yusuf M., Khan S.T., Hayat S. (2018). Zinc oxide nanoparticle-mediated changes in photosynthetic efficiency and antioxidant system of tomato plants. Photosynthetica.

[25] Šebesta M., Kurtinová S., Kolenčík M., Illa R., Faizan M., Hayat S., Yu F. (2021). Enhancement of stress tolerance of crop plants by ZnO nanoparticles. Sustainable Agriculture Reviews 53.

[26] Agarwal S., Jangir L.K., Rathore K.S., Kumar M., Awasthi K. (2019). Morphology-dependent structural and optical properties of ZnO nanostructures. Appl. Phys. A.

[27] Onyszko M., Zywicka A., Wenelska K., Mijowska E. (2022). Revealing the influence of the shape, size, and aspect ratio of ZnO nanoparticles on antibacterial and mechanical performance of cellulose fibers based paper. Part. Part. Syst. Charact..

[28] Tryfon P., Kamou N.N., Pavlou A., Mourdikoudis S., Menkissoglu-Spiroudi U., Dendrinou-Samara C. (2023). Nanocapsules of ZnO nanorods and geraniol as a novel mean for the effective control of *Botrytis cinerea* in tomato and cucumber plants. Plants.

[29] Joksimović A., Arsenov D., Borišev M., Djordjević A., Župunski M., Borišev I. (2025). Foliar application of fullerenol and zinc oxide nanoparticles improves stress resilience in drought-sensitive *Arabidopsis thaliana*. PLoS ONE.

[30] Sun L., Wang Y., Wang R., Wang R., Zhang P., Ju Q., Xu J. (2020). Physiological, transcriptomic, and metabolomic analyses reveal zinc oxide nanoparticles modulate plant growth in tomato. Environ. Sci. Nano.

[31] Nair P.M.G., Chung I.M. (2017). Regulation of morphological, molecular and nutrient status in *Arabidopsis thaliana* seedlings in response to ZnO nanoparticles and Zn ion exposure. Sci. Total Environ..

[32] Wan J., Wang R., Wang R., Ju Q., Wang Y., Xu J. (2019). Comparative physiological and transcriptomic analyses reveal the toxic effects of ZnO nanoparticles on plant growth. Environ. Sci. Technol..

[33] Javed R., Usman M., Yücesan B., Zia M., Gürel E. (2017). Effect of zinc oxide (ZnO) nanoparticles on physiology and steviol glycosides production in micropropagated shoots of *Stevia rebaudiana* Bertoni. Plant Physiol. Biochem..

[34] Gómez-Ortíz N., De la Rosa-García S., González-Gómez W., Soria-Castro M., Quintana P., Oskam G., Ortega-Morales B. (2013). Antifungal coatings based on Ca(OH)_2_ mixed with ZnO/TiO_2_ nanomaterials for protection of limestone monuments. ACS Appl. Mater. Interfaces.

[35] Joshi S., Sabri Y.M., Bhargava S.K., Sunkara M.V., Ippolito S.J. (2021). Band offset in calcium hydroxide mediated CaO-ZnO heterointerfaces. Mater. Sci. Eng. B.

[36] Ashrafi A. (2010). Band offsets at ZnO/SiC heterojunction: Heterointerface in band alignment. Surf. Sci..

[37] Patil B.M., Patil V.L., Bhosale S.R., Bhosale R.R., Ingavale D.R., Patil S.S., Kamble P.D., Bhosale A.G., Mane S.M., Lee J. (2024). Field application of Ca-doped ZnO nanoparticles to maize and wheat plants. Plant Physiol. Biochem..

[38] Thiruvengadam M., Chi H.Y., Kim S.-H. (2024). Impact of nanopollution on plant growth, photosynthesis, toxicity, and metabolism in the agricultural sector: An updated review. Plant Physiol. Biochem..

[39] Tripathi G., Dutta S., Mishra A., Basu S., Gupta V., Kamaraj C. (2025). Nanomaterials impact in phytohormone signaling networks of plants—A critical review. Plant Sci..

[40] Tryfon P., Sperdouli I., Adamakis I.-D.S., Mourdikoudis S., Moustakas M., Dendrinou-Samara C. (2023). Impact of coated zinc oxide nanoparticles on photosystem II of tomato plants. Materials.

[41] Tryfon P., Sperdouli I., Moustaka J., Adamakis I.-D.S., Giannousi K., Dendrinou-Samara C., Moustakas M. (2024). Hormetic Response of Photosystem II Function Induced by Nontoxic Calcium Hydroxide Nanoparticles. Int. J. Mol. Sci..

[42] Moustaka J., Meyling N.V., Hauser T.P. (2021). Induction of a compensatory photosynthetic response mechanism in tomato leaves upon short time feeding by the chewing insect *Spodoptera exigua*. Insects.

[43] Oxborough K., Baker N.R. (1997). Resolving chlorophyll a fluorescence images of photosynthetic efficiency into photochemical andnon-photochemical components—Calculation of qP and Fv′/Fm′ without measuring Fo′. Photosynth. Res..

[44] Moustaka J., Tanou G., Adamakis I.D., Eleftheriou E.P., Moustakas M. (2015). Leaf age dependent photoprotective and antioxidative mechanisms to paraquat-induced oxidative stress in *Arabidopsis thaliana*. Int. J. Mol. Sci..

[45] Giannousi K., Menelaou M., Arvanitidis J., Angelakeris M., Pantazaki A., Dendrinou-Samara C. (2015). Hetero-nanocomposites of magnetic and antifungal nanoparticles as a platform for magneto mechanical stress induction in *Saccharomyces cerevisiae*. J. Mater. Chem. B..

[46] Connorton J.M., Balk J., Rodríguez-Celma J. (2017). Iron homeostasis in plants—A brief overview. Metallomics.

[47] Singh R.P., Handa R., Manchanda G. (2020). Nanoparticles in sustainable agriculture: An emerging opportunity. J. Control. Release..

[48] Harish, Kumar P., Malhotra B., Phalswal P., Khanna P.K., Salim A., Singhal R., Mukhopadhyay A.K. (2020). Effect of reaction rate on the properties of chemically synthesized calcium hydroxide nanoparticles. Mater. Today Proc..

[49] Baharudin K.B., Abdullah N., Derawi D. (2021). Synthesis of raspberry-like structure zinc oxide nanoparticles via glycol-solvothermal, low-temperature solvothermal and coprecipitation methods. C. R. Chim..

[50] Mekuye B., Abera B. (2023). Nanomaterials: An overview of synthesis, classification, characterization, and applications. Nano Select.

[51] Manohar A., Vijayakanth V., Vattikuti S.V.P., Reddy G.R., Kim K.H. (2023). A brief review on Zn-based materials and nanocomposites for supercapacitor applications. J. Energy Storage.

[52] Machín A., Cotto M., Ducongé J., Márquez F. (2023). Artificial photosynthesis: Current advancements and future prospects. Biomimetics.

[53] Pinzari F. (2024). Synthesis, photocatalytic and bio activity of ZnO-TiO_2_ nanocomposites: A review study. Reactions.

[54] Wang X., Xie H., Wang P., Yin H. (2023). Nanoparticles in plants: Uptake, transport and physiological activity in leaf and root. Materials.

[55] Shrestha S., Wang B., Dutta P. (2020). Nanoparticle processing: Understanding and controlling aggregation. Adv. Colloid Interface Sci..

[56] Hu P., An J., Faulkner M.M., Wu H., Li Z., Tian X., Giraldo J.P. (2020). Nanoparticle charge and size control foliar delivery efficiency to plant cells and organelles. ACS Nano.

[57] Krause G.H., Weis E. (1991). Chlorophyll fluorescence and photosynthesis: The basics. Annu. Rev. Plant Physiol. Plant Mol. Biol..

[58] Murchie E.H., Lawson T. (2013). Chlorophyll fluorescence analysis: A guide to good practice and understanding some new applications. J. Exp. Bot..

[59] Guidi L., Calatayud A. (2014). Non-invasive tools to estimate stress-induced changes in photosynthetic performance in plants inhabiting Mediterranean areas. Environ. Exp. Bot..

[60] Kramer D.M., Johnson G., Kiirats O., Edwards G.E. (2004). New fluorescence parameters for the determination of Q_A_ redox state and excitation energy fluxes. Photosynth. Res..

[61] Moustakas M., Sperdouli I., Moustaka J. (2022). Early drought stress warning in plants: Color pictures of photosystem II photochemistry. Climate.

[62] Klughammer C., Schreiber U. (2008). Complementary PS II quantum yields calculated from simple fluorescence parameters measured by PAM fluorometry and the Saturation Pulse method. PAM Appl. Notes.

[63] Kasajima I., Ebana K., Yamamoto T., Takahara K., Yano M., Kawai-Yamada M., Uchimiya H. (2011). Molecular distinction in genetic regulation of nonphotochemical quenching in rice. Proc. Natl. Acad. Sci. USA.

[64] Gawroński P., Witoń D., Vashutina K., Bederska M., Betliński B., Rusaczonek A., Karpiński S. (2014). Mitogen-activated protein kinase 4 is a salicylic acid-independent regulator of growth but not of photosynthesis in Arabidopsis. Mol. Plant.

[65] Moustaka J., Sperdouli I., Panteris E., Adamakis I.-D.S., Moustakas M. (2025). Aspirin foliar spray-induced changes in light energy use efficiency, chloroplast ultrastructure, and ROS generation in tomato. Int. J. Mol. Sci..

[66] Demmig-Adams B., Adams II W.W. (1992). Photoprotection and other responses of plants to high light stress. Annu. Rev. Plant Physiol. Plant Mol. Biol..

[67] Krieger-Liszkay A. (2005). Singlet oxygen production in photosynthesis. J. Exp. Bot..

[68] Ogilby P.R. (2010). Singlet oxygen: There is indeed something new under the sun. Chem. Soc. Rev..

[69] Telfer A. (2014). Singlet oxygen production by PSII under light stress: Mechanism, detection and the protective role of β-carotene. Plant Cell Physiol..

[70] Moustakas M. (2022). Plant photochemistry, reactive oxygen species, and photoprotection. Photochem.

[71] Müller P., Li X.P., Niyogi K.K. (2001). Non-photochemical quenching. A response to excess light energy. Plant Physiol..

[72] Schreiber U., Klughammer C. (2008). Non-photochemical fluorescence quenching and quantum yields in PSI and PSII: Analysis of heat-induced limitations using Maxi-Imaging PAM and Dual-PAM-100. PAM Appl. Notes.

[73] Ruban A.V. (2018). Light harvesting control in plants. FEBS Lett..

[74] Wilson K.E., Ivanov A.G., Öquist G., Grodzinski B., Sarhan F., Huner N.P.A. (2006). Energy balance, organellar redox status, and acclimation to environmental stress. Can. J. Bot..

[75] Zhang J., Li H., Huang X., Xing J., Yao J., Yin T., Jiang J., Wang P., Xu B. (2022). STAYGREEN-mediated chlorophyll a catabolism is critical for photosystem stability during heat-induced leaf senescence in perennial ryegrass. Plant Cell Environ..

[76] Moustakas M., Sperdouli I., Adamakis I.-D.S., Moustaka J., İşgören S., Şaş B. (2022). Harnessing the role of foliar applied salicylic acid in decreasing chlorophyll content to reassess photosystem II photoprotection in crop plants. Int. J. Mol. Sci..

[77] Mittler R. (2017). ROS are good. Trends Plant Sci..

[78] Foyer C.H. (2018). Reactive oxygen species, oxidative signaling and the regulation of photosynthesis. Environ. Exp. Bot..

[79] Moustaka J., Moustakas M. (2023). Early-stage detection of biotic and abiotic stress on plants by chlorophyll fluorescence imaging analysis. Biosensors.

[80] Mittler R., Berkowitz G. (2001). Hydrogen peroxide, a messenger with too many roles?. Redox Rep..

[81] Li H., Jiang X., Lv X., Ahammed G.J., Guo Z., Qi Z., Yu J., Zhou Y. (2019). Tomato *GLR3.3* and *GLR3.5* mediate cold acclimation induced chilling tolerance by regulating apoplastic H_2_O_2_ production and redox homeostasis. Plant Cell Environ..

[82] Foyer C.H. (2020). How plant cells sense the outside world through hydrogen peroxide. Nature.

[83] Moustakas M. (2025). Molecular mechanisms of plant abiotic stress tolerance. Int. J. Mol. Sci..

[84] Kaya C., Ashraf M., Ahmad P., Corpas F.J. (2025). Deciphering the interplay between redox switches and signaling networks in plant stress responses. Crit. Rev. Plant Sci..

[85] Zhang L., Du X., Wang A., Xu Y., Wu T., Liu Z., Shi G., Wei F., Tian B. (2025). *SlGSTU43* and *SlERF-B12* genesare involved in SlMYB15-regulated redox homeostasis to enhance cold tolerance in tomato. J. Agric. Food Chem..

[86] Agathokleous E., Calabrese E.J. (2019). Hormesis can enhance agricultural sustainability in a changing world. Glob. Food Secur..

[87] Moustakas M., Moustaka J., Sperdouli I. (2022). Hormesis in photosystem II: A mechanistic approach. Curr. Opin. Toxicol..

[88] Agathokleous E., Sonne C., Benelli G., Calabrese E.J., Guedes R.N.C. (2023). Low-dose chemical stimulation and pest resistance threaten global crop production. Sci. Total Environ..

[89] Ranjan A., Rajput V.D., Kumari A., Mandzhieva S.S., Sushkova S., Prazdnova E.V., Zargar S.M., Raza A., Minkina T., Chung G. (2022). Nanobionics in crop production: An emerging approach to modulate plant functionalities. Plants.

[90] Pandey K., Dasgupta C.N. (2025). Role of nanobionics to improve the photosynthetic productivity in plants and algae: An emerging approach. 3 Biotech.

[91] Pu S., Yan C., Huang H., Liu S., Deng D. (2019). Toxicity of nano-CuO particles to maize and microbial community largely depends on its bioavailable fractions. Environ. Pollut..

[92] Sun H., Lei C., Xu J., Li R. (2021). Foliar uptake and leaf-to-root translocation of nanoplastics with different coating charge in maize plants. J. Hazard. Mater..

[93] Zhu J., Wang J., Zhan X., Li A., White J.C., Gardea-Torresdey J.L., Xing B. (2021). Role of charge and size in the translocation and distribution of zinc oxide particles in wheat cells. ACS Sustain. Chem. Eng..

[94] Tryfon P., Sperdouli I., Adamakis I.-D.S., Mourdikoudis S., Dendrinou-Samara C., Moustakas M. (2023). Modification of tomato photosystem II photochemistry with engineered zinc oxide nanorods. Plants.

[95] He Q., Zhan J., Liu X., Dong C., Tian D., Fu Q. (2025). Multispectral polarimetric bidirectional reflectance research of plant canopy. Opt. Lasers Eng..

