# Supplementary material for: Short- and Long-Term Effects of Ca(OH)2/ZnO Heteronanostructure on Photosystem II Function and ROS Generation in Tomato
Source: Materials (Basel). 2025 Aug 31;18(17):4078. doi: 10.3390/ma18174078 (PMC12430054; doi:10.3390/ma18174078)
Supplement: Supplementary file 1 [file materials-18-04078-s001.zip › materials-3816684-supplementary.pdf]

materials

MDPI

Short- and Long-term Effects of $Ca(OH)_2$/ZnO Heteronanostructure on Photosystem II Function and ROS Generation in Tomato

Panagiota Tryfon, Julietta Moustaka, Ilektra Sperdouli, Chrysanthi Papoulia, Eleni Pavlidou, George Vourlias, Ioannis-Dimosthenis S. Adamakis, Michael Moustakas and Catherine Dendrinou-Samara

Table S1. Results of two-way ANOVAs testing the effects of *Treatment*, *Time*, and their interaction on photosynthetic parameters measured at 580 and 1000 μmol photons m^{-2} s^{-1}. For each model, the table reports the sum of squares (SS), mean squares (MS), F-statistics, associated p-values, and significance levels. Residuals are included to show model fit and error variance. Significance codes: ***p < 0.001; **p < 0.01; *p < 0.05; .p < 0.1.

Effect	Df	Sum Sq	Mean Sq	F value	Pr(>F)	Signif.	Parameter
Treatment	2	0,4592	0,22962	37,641	2,79E-15	***	ΦPSII 580 μmol photons m^{-2} s^{-1}
Time	1	0,1138	0,11378	18,651	0,0000215	***	
Treatment:Tir	2	0,0528	0,02641	4,329	0,014	*	
Residuals	294	1,7935	0,0061				
Treatment	2	31,96	15,98	19,101	1,59E-08	***	ΦPSII 1000 μmol photons m^{-2} s^{-1}
Time	1	10,13	10,133	12,112	0,000577	***	
Treatment:Tir	2	8,55	4,277	5,112	0,006569	**	
Residuals	294	245,96	0,837				
Treatment	2	74,64	37,32	53,222	2E-16	***	ΦNPQ580 μmol photons m^{-2} s^{-1}
Time	1	12,6	12,6	17,973	0,00003	***	
Treatment:Tir	2	3,27	1,64	2,335	0,0986	.	
Residuals	294	206,17	0,7				
Treatment	2	72,41	36,21	49,377	2E-16	***	ΦNPQ 1000 μmol photons m^{-2} s^{-1}
Time	1	6,34	6,34	8,643	0,00354	**	
Treatment:Tir	2	2,26	1,13	1,54	0,2161		
Residuals	294	215,58	0,73				
Treatment	2	117,02	58,51	98,903	2E-16	***	ΦNO 580 μmol photons m^{-2} s^{-1}
Time	1	1,12	1,12	1,897	0,1695		
Treatment:Tir	2	3,99	1,99	3,369	0,0357	*	
Residuals	294	173,93	0,59				
Treatment	2	117,18	58,59	102,559	2E-16	***	ΦNO 1000 μmol photons m^{-2} s^{-1}
Time	1	0,04	0,04	0,075	0,784723		
Treatment:Tir	2	10,44	5,22	9,139	0,000141	***	
Residuals	294	167,95	0,57				

Effect	Df	Sum Sq	Mean Sq	F value	Pr(>F)	Signif.	Parameter
Treatment	2	10,163	5,082	86,15	2E-16	***	NPQ 580 µmol photons m^{-2} s^{-1}
Time	1	0,764	0,764	12,95	0,000376	***	
Treatment:Tim	2	0,223	0,112	1,89	0,152846		
Residuals	294	17,343	0,059				
Treatment	2	13,78	6,89	111,82	2E-16	***	NPQ 1000 µmol photons m^{-2} s^{-1}
Time	1	0,239	0,239	3,883	0,0497	*	
Treatment:Tim	2	0,271	0,136	2,199	0,1127		
Residuals	294	18,115	0,062				
Treatment	2	74,31	37,16	60,899	2E-16	***	qP 580 µmol photons m^{-2} s^{-1}
Time	1	33,5	33,5	54,903	1,35E-12	***	
Treatment:Tim	2	8,73	4,36	7,153	0,000926	***	
Residuals	294	179,37	0,61				
Treatment	2	47,13	23,563	33,041	1,14E-13	***	qP 1000 µmol photons m^{-2} s^{-1}
Time	1	28,46	28,465	39,915	9,79E-10	***	
Treatment:Tim	2	11	5,499	7,711	0,000545	***	
Residuals	294	209,66	0,713				
Treatment	2	43,66	21,83	26,59	2,43E-11	***	Fv'/Fm' 580 µmol photons m^{-2} s^{-1}
Time	1	7,09	7,087	8,633	0,00356	**	
Treatment:Tim	2	4,72	2,358	2,872	0,0582	.	
Residuals	294	241,37	0,821				
Treatment	2	54,48	27,239	37,23	3,87E-15	***	Fv'/Fm' 1000 µmol photons m^{-2} s^{-1}
Time	1	19,27	19,272	26,341	5,21E-07	***	
Treatment:Tim	2	7,96	3,982	5,442	0,00478	**	
Residuals	294	215,11	0,732				

Effect	Df	Sum Sq	Mean Sq	F value	Pr(>F)	Signif.	Parameter
Treatment	2	0,2818	0,14091	77,854	2E-16	***	1-qL 580 µmol photons m^{-2} s^{-1}
Time	1	0,1186	0,11856	65,509	1,54E-14	***	
Treatment:Tim	2	0,0297	0,01484	8,198	0,000343	***	
Residuals	294	0,5321	0,00181				
Treatment	2	0,1367	0,06836	46,96	2E-16	***	1-qL 1000 µmol photons m^{-2} s^{-1}
Time	1	0,1025	0,10253	70,43	2,04E-15	***	
Treatment:Tim	2	0,0307	0,01535	10,54	0,0000379	***	
Residuals	294	0,428	0,00146				
Treatment	2	55,44	27,721	36,813	5,4E-15	***	ETR 580 µmol photons m^{-2} s^{-1}
Time	1	13,23	13,228	17,566	0,0000367	***	
Treatment:Tim	2	6,63	3,316	4,403	0,0131	*	
Residuals	294	221,39	0,753				
Treatment	2	32,4	16,202	19,377	1,24E-08	***	ETR 1000 µmol photons m^{-2} s^{-1}
Time	1	10,2	10,197	12,195	0,000553	***	
Treatment:Tim	2	8,35	4,176	4,994	0,007365	**	
Residuals	294	245,83	0,836				
Treatment	2	0,1633	0,08163	53,345	2E-16	***	EXC 580 µmol photons m^{-2} s^{-1}
Time	1	0,0531	0,05311	34,709	1,05E-08	***	
Treatment:Tim	2	0,0144	0,00722	4,716	0,00964	**	
Residuals	294	0,4499	0,00153				
Treatment	2	0,0837	0,04187	28,573	4,57E-12	***	EXC 1000 µmol photons m^{-2} s^{-1}
Time	1	0,0314	0,03139	21,419	0,00000554	***	
Treatment:Tim	2	0,0136	0,00679	4,634	0,0104	*	
Residuals	294	0,4308	0,00147				

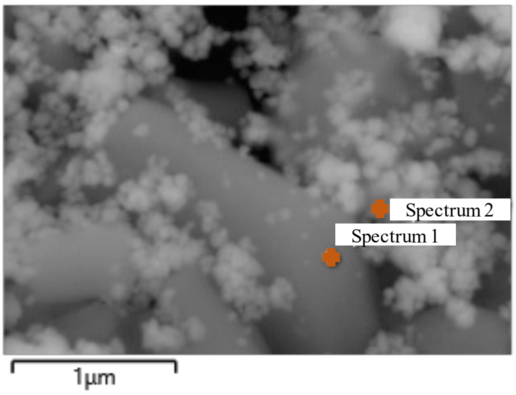

Result Type	Weight %	
Spectrum Label	Spectrum 1	Spectrum 2
Ca	74.01	29.02
Zn	25.99	70.98
Total	100	100

Figure S1. Scanning electron microscopy (SEM) image of CaZnO hetero-nanostructure showcasing spectra one and two, accompanied by their respective compound compositions.

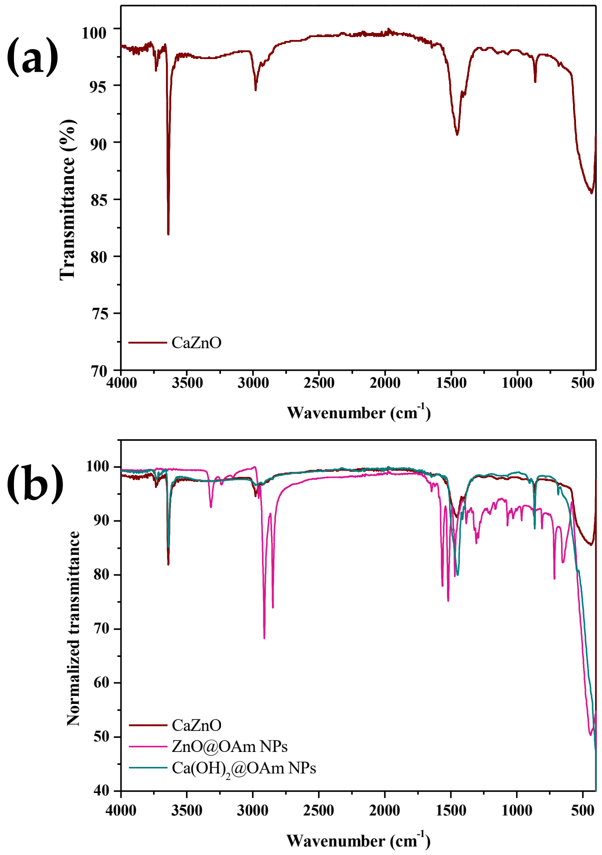

Figure S2. Fourier-transform infrared (FT-IR) spectrum of CaZnO hetero-nanostructure **(a)** and normalized spectra of CaZnO along with the preformed $Ca(OH)_2$@OAm NPs and ZnO@OAm NPs **(b)** [40,41], showing characteristic transmittance peaks indicative of metal-oxygen bonds and organic functional groups.

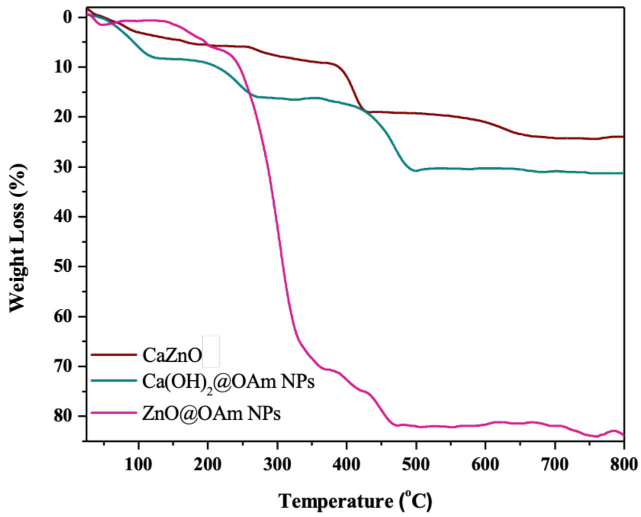

Figure S3. Thermogravimetric analysis (TGA) curves of the newly synthesized CaZnO hetero-nanostructure, in comparison with the individual preformed $Ca(OH)_2$@OAm NPs and ZnO@OAm NPs showing mass loss (%) as a function of temperature (°C). Differences in thermal stability and decomposition stages highlight variations in materials composition.

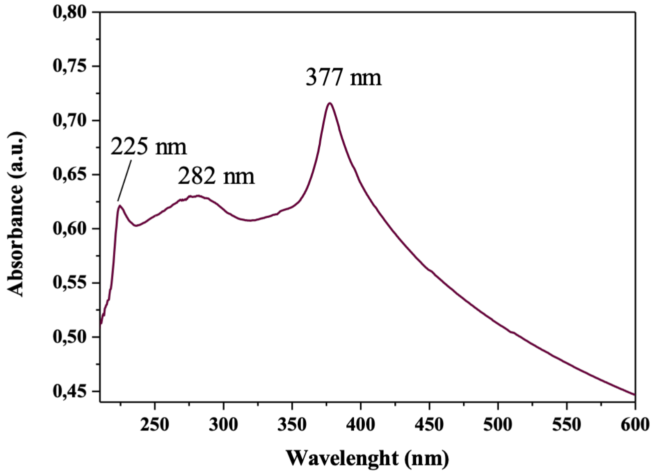

Figure S4. UV-Vis absorbance spectra of CaZnO hetero-nanostructure in aqueous solution over a wavelength range of 210–600 nm.

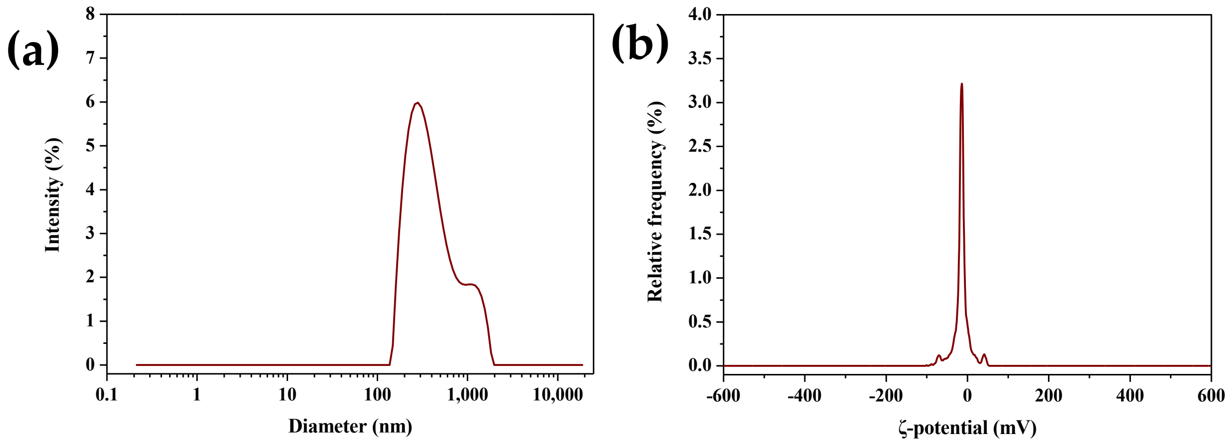

Figure S5. Dynamic light scattering (DLS) analyses of CaZnO hetero-nanostructure: size distribution (**a**) and ζ-potential graph (**b**).
